# Associations between MRI radiomics analysis and tumor-micro milieu in uterine cervical cancer

**DOI:** 10.1007/s00432-025-06253-3

**Published:** 2025-07-21

**Authors:** Hans-Jonas Meyer, Jakob Leonhardi, Anne-Kathrin Höhn, Noura Kabbani, Silke Zimmermann, Jan Borggrefe, Alexey Surov

**Affiliations:** 1 Department of Diagnostic and Interventional Radiology, University Hospital Leipzig, Liebigstraße 20, 04103 Leipzig, Germany; 2 Institute of Pathology, University Hospital of Leipzig, Leipzig, Germany; 3 Department of Gynecology, University Hospital of Leipzig, Leipzig, Germany; 4 Institute of Laboratory Medicine, Clinical Chemistry and Molecular Diagnostics, University of Leipzig, Leipzig, Germany; 5 Institute for Radiology, Neuroradiology and Nuclear Medicine, Johannes Wesling Hospital, Ruhr University Bochum, Minden, Germany

**Keywords:** MRI, Radiomics, Cervical cancer, Tumor-stroma ratio

## Abstract

**Purpose:**

The complex interactions of the tumor micromilieu could be reflected by magnetic resonance imaging (MRI) when analyzed with the radiomics approach. For several tumor entities, it has been shown that radiomics derived from MRI can reflect important characteristics of the tumors. The present study investigated the association radiomics derived from MRI images and histopathological features in uterine cervical cancer.

**Methods:**

The MRI before any treatment was used to extract the radiomics features of T1- and T2-weighted images. The biopsy specimens were stained for Ki 67, e-cadherin, vimentin, programmed-death ligand 1, and tumor-infiltrating lymphocytes (TIL, all CD45 positive cells). Tumor-stroma ratio (TSR) was calculated on routine H&E specimen. Spearman’s correlation analysis and discrimination analyses were performed as statistical analyses.

**Results:**

The patient sample was comprised of 89 female patients with a mean age of 49.3 years ± 14.6 (range 27–77 years) with squamous cell cervical carcinoma. “Kurtosis” derived from T1-weighted images after contrast media application correlated with the Ki-67 index (r = 0.28, *p* = 0.02). “WavEnHL_s-4” derived from T2-weighted images and “S(1.0)Contrast” derived from T1-weighted images after contrast media application showed correlations with TSR (r = − 0.24, *p* = 0.04, each). Several associations were identified between the radiomics features with immune scores defined by programmed-death ligand 1, the highest correlation showed Teta1 derived from T2-weighted images with the combined positive score (r = − 0.38, *p* < 0.01). There were several associations with vimentin expression, the highest showed “Variance” derived from T1-weighted images after contrast media application (r = 0.46, *p* < 0.01).

**Conclusions:**

Radiomics features derived from MRI can reflect tumor characteristics of UCC. Especially immune-related features were reflected by the MRI texture features. Proliferation potential, composition of the extracellular matrix and tumor-stroma ratio were also significantly associated with radiomics features. These presented results need to be evaluated in an independent cohort to test their stability.

## Introduction

Uterine cervical cancer (UCC) is the eighth most prevalent cancer and the ninth leading cause of cancer death in women worldwide (Bray et al. 2022). Due to its exceptional soft tissue contrast (Haldorsen et al. 2019), magnetic resonance imaging (MRI) is the preferred imaging method for UCC staging. It is well known that MRI cannot only be used for diagnostic staging but also provide prognostic factors in patients with UCC (Ytre-Hauge et al. 2018; Surov et al. 2017; Bizzarri et al. 2023).

Among the novel imaging analyses techniques, radiomics is a new quantitative imaging analysis to provide novel prognostic imaging markers. With a total of 57 published papers radiomics analyses demonstrated promising results in the field of UCC as shown in a recent systematic review (Bizzarri et al. 2023).

MRI radiomics showed high diagnostic accuracy to predict treatment outcomes in patients undergoing radiotherapy and chemotherapy in both curative and palliative settings across numerous independent patient cohorts with diverse investigated MRI radiomics signatures (Bizzarri et al. 2023; Manganaro et al. 2021).

Whereas the macroscopic scale of MRI can reflect tumorbiology in UCC, there is clear evidence that the microscopic scale with various immunohistochemical features, such as epidermal growth factor, hypoxia-inducible factor 1alpha, vascular endothelial growth factor, human epidermal growth factor receptor 2 (HER 2), and histone 3, are linked to prognosis and treatment outcome prediction (Gadducci et al. 2013; Soonthornthum et al. 2011; Noordhuis et al. 2009; Kim et al. 2002; Liu et al. 2014; Rocha Martins et al. 2023). The tumor microenvironment's predictive and prognostic function in determining the composition of the tumor in terms of tumor-stroma ratio and tumoral infiltrating lymphocytes in UCC has been demonstrated in recent studies (Liu et al. 2014; Rocha Martins et al. 2023).

A first study tried to cross the scales between imaging and histology and demonstrated associations between histogram features derived from T1- and T2-weighted images with Her 2 status and EGFR expression in UCC in a preliminary study (Meyer et al. 2019).

The heterogeneity of the tumor composition on histology may result in different MRI texture features reflected by radiomics analyses. There is definite need for novel imaging analyses to characterize the tumors in a more comprehensive way.

Based on these previous analyses, it seems plausible that imaging could non-invasively predict the malignancy potential as well as some characteristics of the underlying tumor microstructure. However, comprehensive analyses are needed to explore the complex interactions between imaging and histopathology.

Nevertheless, there is still few data investigating the direct spatial characteristics between MRI radiomics features and the underlying histopathologic features in UCC.

Consequently, the aim of the present study was to explore potential connections between radiomic characteristics obtained from MRI and histological characteristics such as the expression of Ki 67, extracellular matrix features, tumor-stroma ratio, and tumor-infiltrating lymphocytes in UCC.

## Material and methods

### Patient acquisition

All patients with UCC who presented consecutively to our tertiary referral hospital were evaluated retrospectively. In line with the ethical standards of the institutional and/or national research committee and the 1964 Helsinki declaration and its later amendments or comparable ethical standards (Ethical code: 012/13–28012013), the study was carried out after obtaining clearance from the local ethics committee.

The inclusion criteria were biopsy-confirmed squamous cell UCC. The MRI had to be performed before the biopsy and any form of treatment. The exclusion criteria included artefacts of the MRI images and missing biopsy specimens.

Squamous cell cervical cancer affected 89 female patients with an average age of 49. 3 years ± 14. 6 (range 27–77 years) in the patient sample.

### Magnetic resonance imaging

In every instance, a 1.5 T scanner was used to conduct the pelvic MRI (Aera, Siemens, Erlangen, Germany). Our investigation procedure included the following sequences: an axial T2 weighted (T2w) turbo spin echo (TSE) sequence (TR/TE: 5590/105), a sagittal T2w TSE sequence (TR/TE: 4110/131), an axial T1 weighted (T1w) TSE sequence (TR/TE:1310/12), an axial T1 TSE sequence following intravenous administration of contrast medium (0. 1 mmol/kg body weight Gadobutrol, Bayer Healthcare, Germany) (TR/TE:912/12), and a sagittal post contrast T1 TSE (TR/TE: 593/12). DWI was carried out using a multishot SEEPI sequence (b 0 and b 1000 s/mm^2^, repetition time: 4900 ms; echo time: 105 ms; slice thickness: 5 mm; matrix: 88 × 134; field of view: 450 × 450 mm.

### MRI texture analysis

This investigation only included MR images taken before the biopsy. The area of interest underwent texture analysis using MaZda software (version 4. 7, available at http://www. eletel. p. lodz. pl/mazda/) (Strzelecki et al. 2013; Szczypiński et al. 2009). The axial T1-weighted images (with and without contrast media application) and the T2-weighted images were examined. For each sequence, a total of 279 texture characteristics were retrieved, totalling 837 texture features for each patient.

In accordance with techniques used in similar texture analysis research (Meyer et al. 2017), gray-level normalization was performed for each ROI by limiting dynamics to μ ± 3 standard deviations, thereby reducing the effect of fluctuations in contrast and brightness.

Region of interest (ROI) delineation was conducted by an experienced radiologist, blinded to histopathological results, with more than four years of experience. To do so, the representative MR-images with maximal depiction of the tumors were retrieved in DICOM format. A polygonal ROI was then placed covering the cross section of the tumor in one axial slice. Tumor edges were spared out to prevent partial volume effects.

**Fig. 1:**
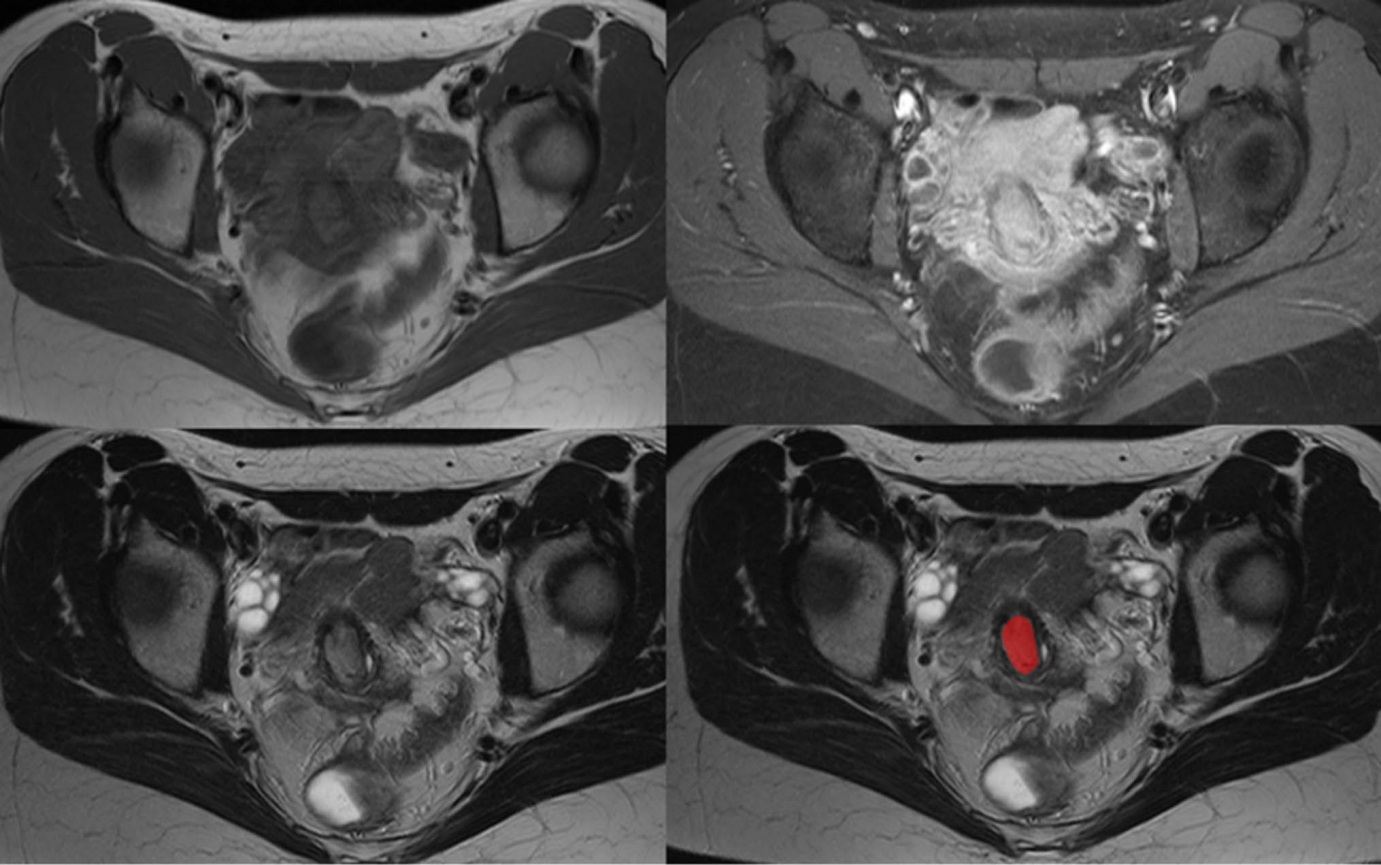
Representative patient of the present cohort, FIGO IB1, T1b1 N0 M0, G1. **A.** T1-weighted axial image showing the small inhomogeneous tumor with intraluminal extension. **B**. T1-weighted axial image contrast media application with fat-saturation. The tumor shows a remarkable contrast media uptake **C.** T2-weighted axial sequence. **D.** Drawn region of interest within the boundaries of the tumor displayed in red

The measurement is shown using a typical example from the current cohort in Fig. 1.

To minimize the risk of overfitting and multicollinearity, feature reduction was applied. Only texture features with pairwise correlation coefficients below 0.7—indicating statistical independence—were retained for further analysis.

For a total number of n = 30 randomly chosen patients, a second read was performed by as second radiologist with more than 8 years of experience, to investigate inter reader agreement. This was done with the calculation of intraclass correlation coefficients. The inter-reader agreement was evaluated for the independently statistically significant texture features and was conducted for T2-weighted as well as T1-weighted images without and after contrast media application.

### Histopathological analysis

A board-certified pathologist (AKH) carried out the histopathology examination without knowing the patients' identities or any imaging information. After being formally fixed and embedded in paraffin, the tissue was serially sectioned (2 µm), dewaxed in xylol, and rehydrated using a series of decreasing ethanol concentrations. Immunohistochemistry and conventional hematoxylin and eosin (HE) staining were carried out for each sample. Before counterstaining with HE solution, we used the automated immunohistochemistry slide staining system VENTANA BenchMark ULTRA (Roche Diagnostics GmbH) and the VENTANA iVIEW DAB Detection Kit (Roche Diagnostics GmbH) for antigen detection. Antigen retrieval was carried out using CC1mild, then the tissue was incubated at 36 °C for 32 min with certain primary antibodies that recognized CD45/leucocyte common antigen (polyclonal mouse antibody, clone 2B11 + PD7/26; DAKO/ Agilent M0701) or Ki 67 (polyclonal mouse antibody, clone Mib1; DAKO/Agilent M7240), diluted at a ratio of 1:500 or 1:100, respectively. All histological features were evaluated in five power fields (× 40; 0. 23 mm2 per field). The mean values of the quantified parameter were calculated for each specimen. The tumor-stroma ratio (TSR) was assessed on the HE-stained sample, and the percentages of tumor and stroma material were given separately. Tumor-infiltrating immune cell density was estimated as the average of total cell counts or CD45 + leukocytes per high power field, respectively. The proportion of stained HPF was determined using vimentin (DAKO, clone Vim 3B4, dilution 1:200) to stain the extracellular matrix. The mouse Anti-E-cadherin monoclonal primary antibody (Clone: NCH38; M3612; DakoCytomation, Denmark) was used to stain for E-cadherin. The percentage of Ki 67-positive cells out of all tumor cells, known as the Ki 67 index, showed the rate of proliferation. The highest value of the five measurements was used to define Ki 67. The Nikon ECLIPSE NiE microscope was used for histopathological examination.

### Statistical analysis

SPSS (IBM, Version 25. 0; Armonk, NY, USA) was used for the statistical analysis and graphical generation. Descriptive statistics (absolute and relative frequencies) were used to assess the collected data. After testing for normality distribution, Spearman's correlation coefficient (r) was used to analyze relationships between the examined radiomics features and the examined histopathological parameters. Group differences were determined using the Mann Whitney U-test. Inter-reader variability was assessed using intraclass correlation coefficient (ICC) calculations, considering levels of 0.5–0.75 as moderate, between 0.75 and 0.90 as good and above 0.90 as excellent. Radiomic features with ICC—values of below 0.6 were excluded from further analysis.

In every case, p values below 0.05 were used to indicate statistical significance.

## Results

Table 1 summarizes the characteristics of the patient sample.

**Table 1:** Overview of the investigated patient sample (n = 89)

Parameter	Number (%)
Age	mean age of 49.3 years ± 14.6 (range 27–77)
*T stage*	
1	39 (43.8%)
2	37 (41.6%)
3	8 (9.0%)
4	5 (5.6%)
*N stage*	
N0	46 (51.7%)
N +	43 (48.3%)
*M stage*	
M0	76 (85.4%)
M1	13 (14.6%)
*Tumor grading*	
G1	8 (9.0%)
G2	38 (42.7%)
G3	43 (48.3%)
*FIGO classification*	
IA	1 (1.1%)
IB1	28 (31.5%)
IB2	5 (5.6%)
IIA	5 (5.6%)
IIB	27 (30.3%)
IIIA	3 (3.4%)
IIIB	14 (15.7%)
IVA	4 (4.5%)
IVB	2 (2.2%)

The cohort was predominantly comprised of early tumors with n = 39 (43.8%) T1 tumors and n = 37 (41.6%) T2 tumors, respectively. Higher tumor stages were consequently rare.

Nodal negative stage was found in n = 46 (51.7%) cases and n = 43 (48.3%) were nodal positive. A total of 13 patients already showed distant metastases (14.6%).

Regarding grading, most tumors were moderate and poorly differentiated (n = 38, 32.7% and n = 43, 48.3%, respectively).

### Inter-reader variability

Inter-reader variability analysis showed a strong correlation between both reads across most independent texture features, ranging from 0.63 to 0.99 (for T2-weighted images), 0.67–0.98 (for T1-weighted images without contrast) and 0.75–0.99 (for T1-weighted images after contrast media application).

For a full overview of the remaining independent texture features with ICC > 0.6, see Tables 2, 3, 4.

**Table 2:** Results of the interreader agreement with intraclass coefficient analysis for the T2-derived features

Texture parameter	ICC	95% CI	*p*
_MinNorm	0.97	0.94–0.99	< 0.001
_MaxNorm	0.99	0.98–0.99	< 0.001
Variance	0.97	0.94–0.99	< 0.001
Skewness	0.82	0.62–0.91	< 0.001
Kurtosis	0.84	0.67–0.93	< 0.001
S(1.0)AngScMom	0.86	0.72–0.94	< 0.001
S(1.0)Contrast	0.93	0.84–0.97	< 0.001
GrSkewness	0.63	0.24–0.82	0.004
Teta1	0.92	0.84–0.96	< 0.001
WavEnLH_s-4	0.81	0.60–0.91	< 0.001
WavEnHL_s-4	0.73	0.42–0.88	< 0.001
WavEnHH_s-4	0.81	0.60–0.91	< 0.001

**Table 3:** Results of the interreader agreement with intraclass coefficient analysis for the T1-derived parameters without contrast media

Texture parameter	ICC	95% CI	*p*
_MinNorm	0.98	0.96–0.99	< 0.001
Variance	0.80	0.58–0.90	< 0.001
Skewness	0.92	0.83–0.96	< 0.001
Kurtosis	0.97	0.94–0.99	< 0.001
S(1.0)AngScMom	0.97	0.93–0.99	< 0.001
S(1.0)Contrast	0.87	0.72–0.94	< 0.001
S(1.0)InvDfMom	0.96	0.91–0.98	< 0.001
GrSkewness	0.82	0.62–0.91	< 0.001
WavEnLH_s-4	0.93	0.86–0.97	< 0.001
WavEnHH_s-4	0.88	0.74–0.94	< 0.001
WavEnLH_s-5	0.67	0.31–0.85	0.001
WavEnHL_s-5	0.75	0.48–0.88	< 0.001
WavEnHH_s-5	0.84	0.66–0.92	< 0.001

**Table 4:** Results of the interreader agreement with intraclass coefficient analysis for the T1-derived features after contrast media application

Texture parameter	ICC	95% CI	*p*
_MinNorm	0.99	0.97–0.99	< 0.001
_MaxNorm	0.99	0.97–0.99	< 0.001
Variance	0.98	0.96–0.99	< 0.001
Skewness	0.75	0.47–0.88	< 0.001
Kurtosis	0.79	0.56–0.90	< 0.001
S(1.0)AngScMom	0.96	0.92–0.98	< 0.001
S(1.0)Contrast	0.98	0.96–0.99	< 0.001
Teta1	0.94	0.87–0.97	< 0.001
Teta2	0.98	0.95–0.99	< 0.001
WavEnHL_s-4	0.93	0.86–0.97	< 0.001

### Correlation analysis with T-, N-stage and tumor grading

There were some correlations between the investigated radiomics features and T-stage.

For the T2-weighted features, the parameter “WavEnLH_s-4” (r = − 0.28, *p* = 0.01) was statistically significant correlated with T-stage. For the T1-weighted features after contrast media application it was “S(1.0)AngScMom.2” showed a correlation with T-stage (r = 0.27, *p* = 0.01). However, no radiomics features derived from native T1-weighted images was associated with T-stage. The results are presented in Table 5.

**Table 5:** Correlation analysis with T-stage

Texture features	Spearman ‘s r	*p*-value
*T2 -weighted*		
S(1.0)SumOfSqs	− 0.30	< 0.01
WavEnLH_s-4	− 0.28	0.01
WavEnHH_s-4	− 0.25	0.03
*T1-weighted after contrast media*		
S(1.0)AngScMom.2	0.27	0.01
S(1.0)Contrast.2	− 0.25	0.03
Teta2	0.26	0.02

The results for the associations with tumor grading are provided by Table 6. “GrSkewness” derived from the T1-weighted features without contrast media application showed the highest correlation with tumor grading (r = − 0.27, *p* = 0.01).

**Table 6:** Correlation analysis with tumor grading

Texture features	Spearman ‘s r	*p*-value
*T2-weighted*		
S(1.0)AngScMom	0.25	0.02
GrSkewness	− 0.23	0.04
*T1-weighted without contrast media*		
GrSkewness.1	− 0.27	0.01
WavEnHH_s-5	0.24	0.03

In discrimination analysis, there were statistically significant differences in regard of the N stage. The highest significance was reached by “WavEnHH_s-4” derived from T2-weighted images (*p* = 0.03). These results are presented in Table 7.

**Table 7:** Discrimination analysis for N stage

Texture feature	N0	N +	*p*-value
*T2-weighted*			
Kurtosis	0.12 ± 0.88	0.39 ± 0.85	0.04
WavEnLH_s-4	387.63 ± 162.44	312.29 ± 151.51	0.04
WavEnHH_s-4	125.84 ± 132.40	84.10 ± 48.81	0.03
*T1-weighted features after contrast media application*			
S(1.0)SumOfSqs	106.46 ± 3.17	107.62 ± 2.67	0.03
Teta2	− 0.78 ± 0.05	− 0.73 ± 0.09	0.03

### Correlation analysis with proliferation potential, vimentin and e-cadherin expression

**Fig. 2:**
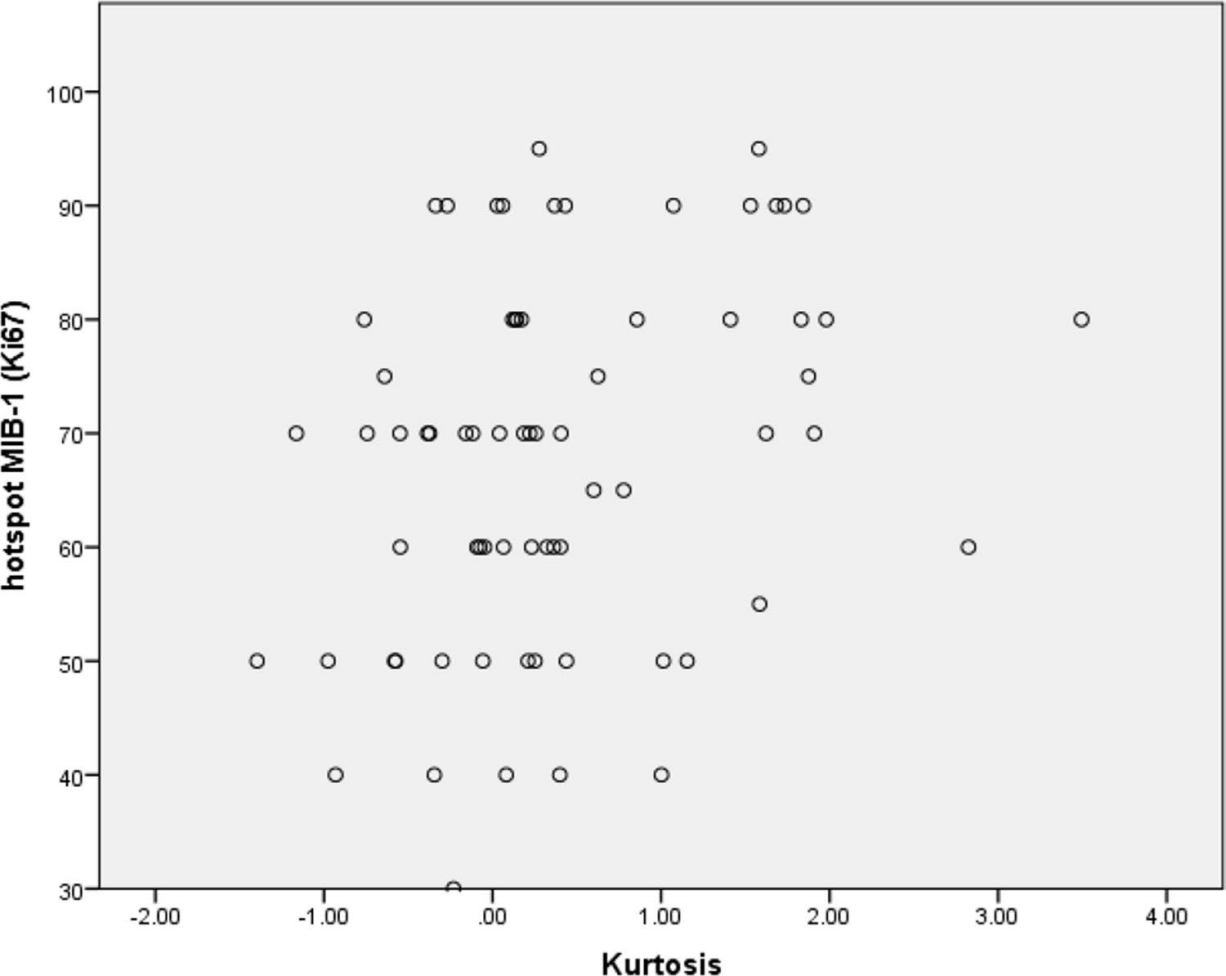
Spearman’s correlation analysis between “Kurtosis” derived from T1-weighted images after contrast media application with Ki-67 index (r = 0.28, *p* = 0.02)

Only the radiomics feature “Kurtosis”, derived from T1-weighted images after contrast media application, was statistically significantly correlated with the proliferation index Ki-67 (r = 0.28, *p* = 0.02, Fig. 2).

### Correlation analysis with tumor-stroma ratio

Two radiomics features showed correlations with TSR: “WavEnHL_s-4” derived from T2-weighted images r = − 0.24, *p* = 0.04) and “S(1.0)Contrast” derived from T1-weighted images after contrast media application (r = − 0.24, *p* = 0.04).

### Correlation analysis with vimentin expression

**Fig. 3:**
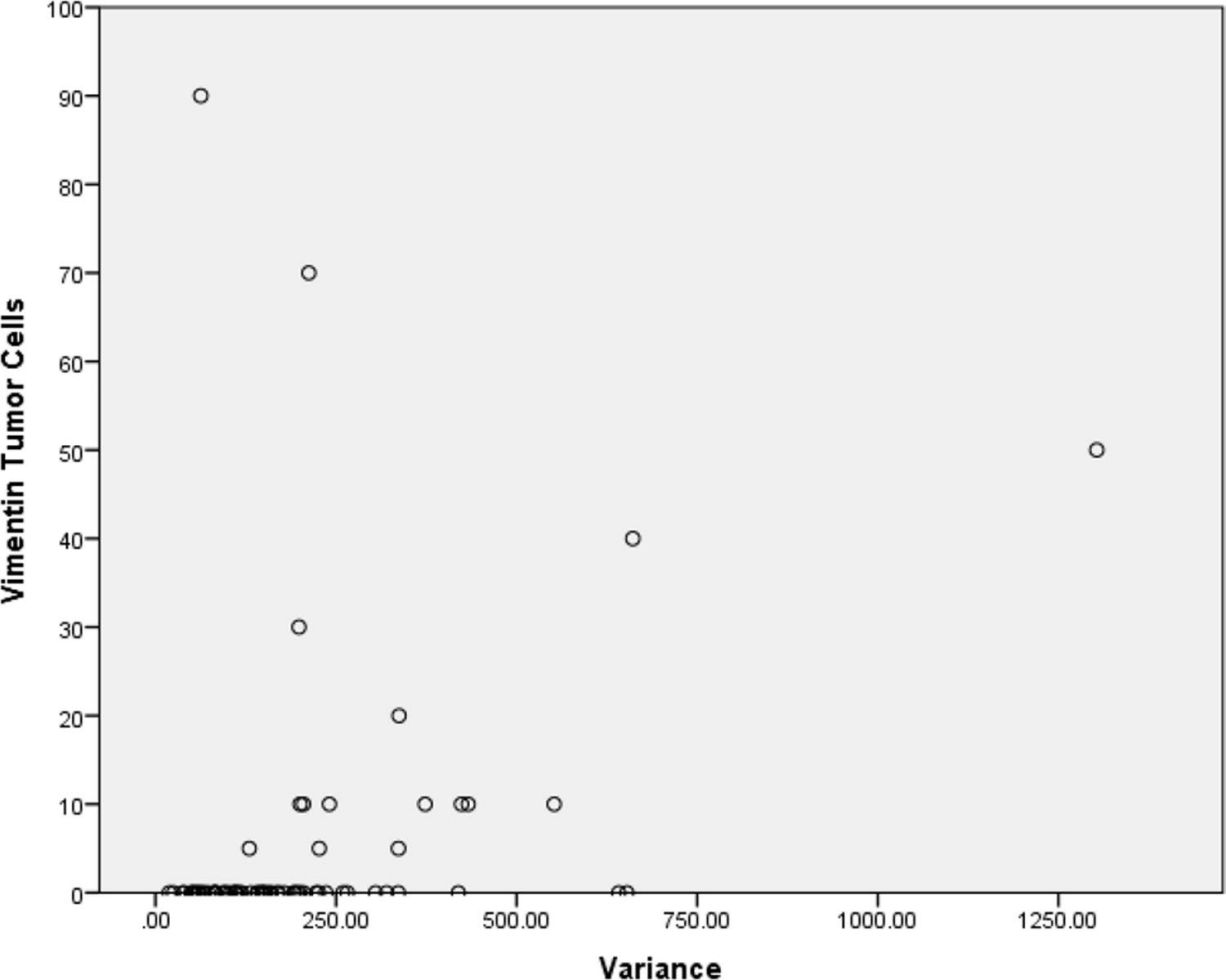
Spearman’s correlation analysis between “Variance” derived from T1-weighted images after contrast media application with vimentin expression (r = 0.46, *p* < 0.01)

There were several associations with vimentin expression, the highest showed “Variance” derived from T1-weighted images after contrast media application (r = 0.46, *p* < 0.01, Fig. 3). These associations with vimentin are presented in Table 8.

**Table 8:** Correlations with vimentin expression

Texture features	Spearman ‘s r	*p*-value
*T2-weighted*		
GrSkewness	0.29	0.02
*T1-weighted without contrast media*		
GrSkewness	0.26	0.03
*T1-weighted after contrast media application*		
_MaxNorm	0.399	< 0.01
Variance	0.46	< 0.01

### Correlation analysis with and e-cadherin

**Fig. 4:**
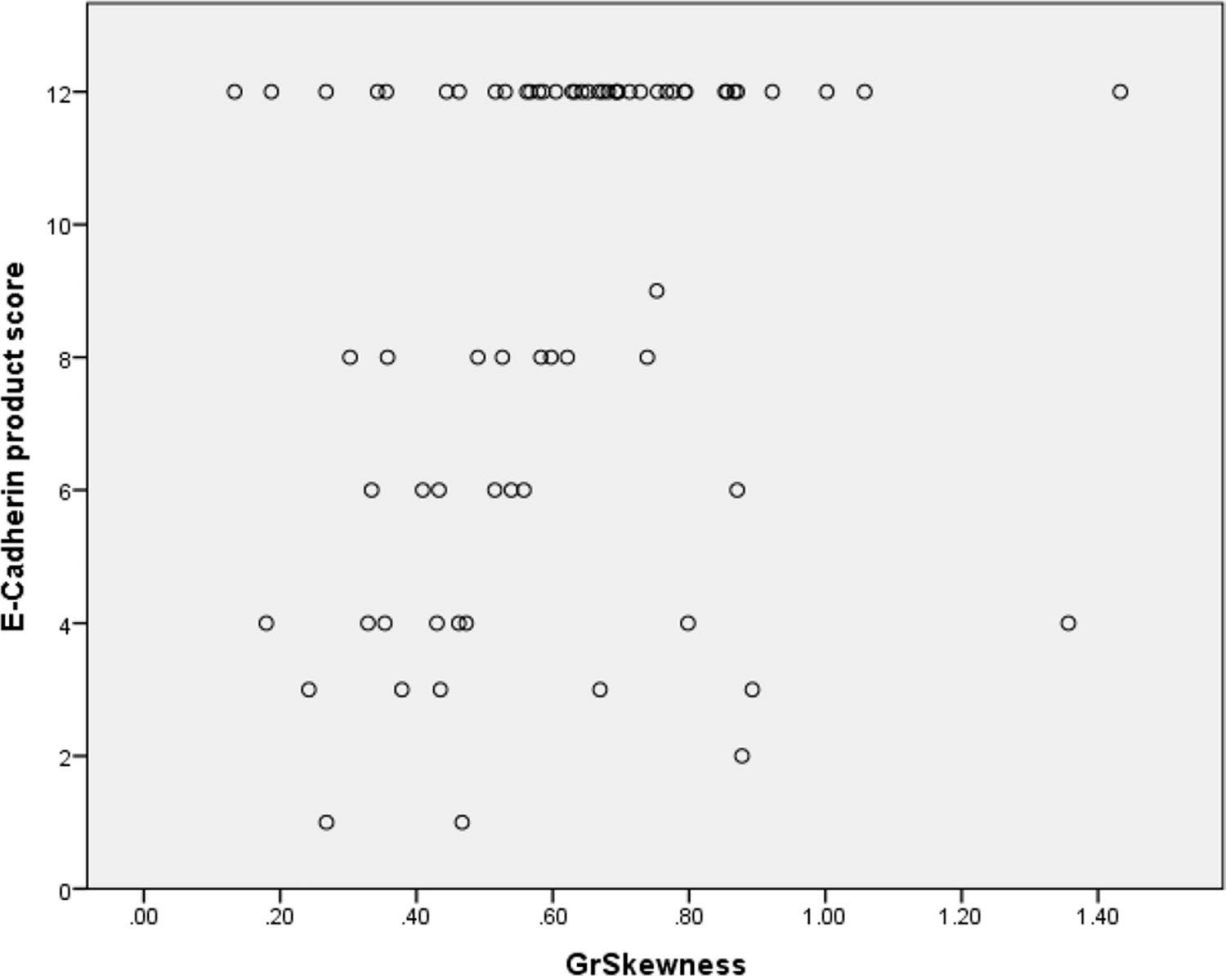
Spearman’s correlation analysis between GrSkewness derived from T2-weighted images with e-cadherin-expression (r = 0.32, *p* < 0.01)

Regarding e-cadherin, there were also several statistically significant associations, the highest reached “_MinNorm” derived from T1-weighted images after contrast media application with r = − 0.34, *p* < 0.01) and “GrSkewness” derived from T2-weighted images (r = 0.32, *p* < 0.01) (Fig. 4). These results are highlighted in Table 9.

**Table 9:** Correlations with e-cadherin expression

Texture features	Spearman ‘s r	*p*-value
*T2-weighted*		
GrSkewness	0.32	< 0.01
*T1-weighted after contrast media application*		
_MinNorm	− 0.34	< 0.01
Variance	0.32	< 0.01
S(1.0)Contrast	− 0.24	0.04
Teta1	0.26	0.03

### Correlation analysis with PD-L1 scores and tumor-infiltrating lymphocytes

Several radiomics features correlated with the different PD-L1 scores, the highest correlation showed “Teta1” derived from T2-weighted images with the combined positive score (r = − 0.38, *p* < 0.01). The correlations with the immune scores are presented in Table 10.

**Table 10:** Correlation analysis with the different PD-L1 scores

Texture features	Spearman ‘s r	*p*-value
*Tumor positive score (TPS)*		
*T2-weighted*		
Teta1	− 0.35	< 0.01
WavEnHL_s-4	0.25	0.04
*Immune cell positive score (ICS)*		
*T2-weighted*		
WavEnHH_s-4	− 0.25	0.03
*Combined positive Score (CPS)*		
*T2-weighted*		
Teta1	− 0.38	< 0.01
*T1-weighted without contrast media*		
WavEnLH_s-4.1	− 0.22	0.04

Regarding the tumor- and stroma infiltrating lymphocytes, “S(1.0)Contrast” derived from T1-weighted images without contrast media showed the strongest correlation with the tumor-infiltrating lymphocytes (r = − 0.30, *p* = 0.01). The other statistically significant correlations are shown in Table 11.

**Table 11:** Correlation analysis with tumor- and stroma infiltrating lymphocytes

Texture features	Spearman ‘s r	*p*-value
*Stroma-infiltrating lymphocytes*		
*T2-weighted*		
Teta1	− 0.25	0.04
*T1-weighted without contrast media*		
WavEnHH_s-4	− 0.29	0.01
WavEnLH_s-5	− 0.28	0.02
WavEnHH_s-5	− 0.28	0.02
*T1-weighted after contrast media application*		
WavEnHL_s-4	− 0.26	0.03
*Tumor-infiltrating lymphocytes*		
*T2-weighted*		
Teta1	− 0.24	0.04
*T1-weighted without contrast media*		
S(1.0)Contrast	− 0.30	0.01
WavEnHH_s-4	− 0.26	0.03
*T1-weighted after contrast media application*		
Teta2	0.27	0.02

## Discussion

This present study elucidated the complex interactions between MRI radiomics features and histopathological features in UCC. The key findings of the study are the correlations between imaging parameters and tumor aspects including proliferation potential, extracellular matrix, tumor-stroma ratio, immune scores and tumor-infiltrating lymphocytes. Different radiomics features derived from T1- and T2-weighted images were sensitive for the underlying tumor microstructure and could reflect different aspects of tumor biology.

The present results are strengthening the ever-growing evidence that the imaging phenotype can predict the underlying histopathology. Notably, the identified associations are not as strong that they could predict the histopathology in highly accurate way and could replace histopathology in the near future. However, the results can be considered as promising for further studies to better understand the complex interactions between imaging phenotype and histology and to further explore which histopathology features may be sufficiently predictable by imaging and which are not.

To change clinical decision making, there should be a very high diagnostic accuracy to correctly predict the histopathology feature. Only in certain circumstances it would be justified to perform the surrogate histopathology assessment by radiomics analysis. A more comprehensive, multi-sequence prediction model could lead to higher diagnostic accuracy to better characterize the tumors in a non-invasive way.

This could also be interesting in patients undergoing treatment as MRI could provide quantitative measures in clinical routine, whereas repetitive biopsy analyses are not suitable in most cases. In addition to that, MRI could provide insight of the whole tumor, whereas the biopsy specimen can only provide insight into a small area of the tumor.

The presented results are based upon the rationale that MRI imaging markers are correlated with cell density and different histopathological features of tumors, as was demonstrated in various tumor entities (Meyer et al. 2017; Meyer et al. 2022; Yin et al. 2024; Just 2014; Schob et al. 2017).

The prognostic role of MRI radiomics in UCC is well established (Zhao et al. 2022; Wang et al. 2022). An interesting study from China investigated 57 patients with a comprehensive radiomics approach to correctly classify the early tumor stages FIGO Ib from IIa (Zhao et al. 2022). The identified radiomics signature was based upon T2-weighted and DWI-derived sequences and achieved a very good AUC of 0.907. Our present results can also indicate that MRI radiomics showed associations with T stages. Presumably, the higher stages show more heterogeneity of the underlying microstructure, which can be assessed by the imaging markers.

Similar results regarding nodal status and discrimination between the histological subtype were demonstrated in a multicenter study to also ensure the stability of the analysis (Liu et al. 2023).

First interesting studies can also show the predictive role of MRI radiomics with a prediction of the overall survival after 3- and 5-years respectively in patients undergoing radiotherapy and chemotherapy (Li et al. 2023; Wei et al. 2022; Gui et al. 2021; Cai et al. 2024; Zheng et al. 2022).

The study by Chong et al. showed that MRI radiomics could predict tumor budding status of the tumors, which is the presence of a single cancer cell or clusters of up to four cancer cells at the invasive tumor front or within the main tumor body (Chong et al. 2021). This study demonstrated that MRI radiomics is able to reflect even distinctive differences of tumor composition, which may be one good explanation for the identified correlations in the present study.

Deng et al. could demonstrate the possibility to predict lymph node metastasis and even VEGF expression of the tumors using a MRI signature derived from T1- and T2-weighted images (Deng et al. 2021).

It was demonstrated that T2-derived skewness may be able to discriminate Her 2- positive from negative tumors and that several T1-weighted features were associated with EGFR expression of the tumors (Meyer et al. 2019). One can clearly deduce from these results that different sequences harbor different potential to predict histopathology features. In addition, this makes it clear that the MRI radiomics signature must be built from different MRI sequences to fully exploit the potential of non-invasive prediction of histopathology features.

The prognostic relevance was demonstrated for all the investigated immunohistochemical parameters, which reflects the proliferation potential, composition of the tumor-stroma and extracellular matrix and the amount of tumor infiltrating lymphocytes (Gadducci et al. 2013; Soonthornthum et al. 2011; Noordhuis et al. 2009; Kim et al. 2002; Liu et al. 2014; Rocha Martins et al. 2023). The outlook of these identified associations may be a better treatment guidance, especially for immunotherapy. Presumably, MRI radiomics could better detect ongoing tumor changes during immunotherapy than conventional size measurements and could therefore change clinical care in this regard. However, there are a lot of research efforts needed to confirm this assumptive role of MRI radiomics.

The merit of the present study is that several statistical associations between different MRI texture features and different histopathological features of UCC were demonstrated. However, these must be validated in an independent patient cohort to test for stability and external validity. Only when this is also performed, a potential translation into clinical routine can be performed to test these non-invasive tumor characteristics in practice.

The present study is not free from limitations. First, it is a retrospective study with known inherent bias. However, the imaging and pathological analysis was performed independently and blinded to each other to reduce possible bias. Another important aspect is that possible spatial differences may be present between the biopsy analysis, which covers only a small part of the tumor and the MRI segmentation, which represents the whole tumor. There may be differences between imaging and histology, especially in heterogeneous tumors. However, due to the present study design, there is no possible to address this fact in a sufficient way. Second, the patient sample is comprised from a single center, which could limit possible translation to other MRI scanners and used imaging sequences. Therefore, further confirmation studies are needed to validate the presented results. Third, the present analysis did not include the diffusion-weighted imaging (DWI) sequence, which could reflect more tissue characteristics of UCC. The sequence was not further analyzed as not every patient was investigate with DWI. Forth, a more comprehensive preprocessing with noise reduction techniques could have resulted in different results. For a possible translation of the present texture features to different MRI scanners, there is need for a further harmonization between the different MRI sequences.

In conclusion, radiomics features derived from MRI can reflect tumor characteristics of UCC. Especially immune-related features were reflected by the MRI texture features. Proliferation potential, composition of the extracellular matrix and tumor-stroma ratio were also significantly associated with radiomics features. These present results need to be evaluated in an independent cohort to test their stability.

## Data Availability

No datasets were generated or analysed during the current study.
